# The Female Reproductive Tract Microbiome—Implications for Gynecologic Cancers and Personalized Medicine

**DOI:** 10.3390/jpm11060546

**Published:** 2021-06-11

**Authors:** Anthony E. Rizzo, Jennifer C. Gordon, Alicia R. Berard, Adam D. Burgener, Stefanie Avril

**Affiliations:** 1 Department of Obstetrics and Gynecology, Division of Gynecologic Oncology, University Hospitals Cleveland Medical Center and Case Western Reserve University, Cleveland, OH 44106, USA; Anthony.Rizzo2@UHhospitals.org (A.E.R.); Jennifer.Gordon@UHhospitals.org (J.C.G.); 2 Department of Obstetrics and Gynecology, University of Manitoba, Winnipeg, MB R3E 0W2, Canada; alicia.berard@case.edu; 3 Center for Global Health and Diseases, Case Western Reserve University, Cleveland, OH 44106, USA; 4 Case Comprehensive Cancer Center, Cleveland, OH 44106, USA; 5 Department of Pathology, Case Western Reserve University and University Hospitals Cleveland Medical Center, Cleveland, OH 44106, USA

**Keywords:** female reproductive tract microbiome, upper reproductive tract microbiome, vaginal microbiome, uterine microbiome, gynecologic cancer, endometrial cancer, ovarian cancer

## Abstract

The microbial colonization of the lower female reproductive tract has been extensively studied over the past few decades. In contrast, the upper female reproductive tract including the uterine cavity and peritoneum where the ovaries and fallopian tubes reside were traditionally assumed to be sterile under non-pathologic conditions. However, recent studies applying next-generation sequencing of the bacterial 16S ribosomal RNA gene have provided convincing evidence for the existence of an upper female reproductive tract microbiome. While the vaginal microbiome and its importance for reproductive health outcomes has been extensively studied, the microbiome of the upper female reproductive tract and its relevance for gynecologic cancers has been less studied and will be the focus of this article. This targeted review summarizes the pertinent literature on the female reproductive tract microbiome in gynecologic malignancies and its anticipated role in future research and clinical applications in personalized medicine.

## 1. Introduction

The human microbiome refers to the collective genomes of bacteria, viruses, bacteriophages, protozoa, and fungi that symbiotically inhabit the human body. While microorganisms make up only about 1 to 3 percent of the human body mass, microbial cells outnumber human cells by an estimated factor of ten [1]. The Human Microbiome Project, a conceptual extension of the Human Genome Project [1,2], has been increasing the understanding of the microbiome’s dynamic role in influencing inflammation, metabolic and cellular pathways, mucosal homeostasis, and host immune responses throughout the human body [3] as well as its perturbation in multiple types of human diseases including neoplastic processes. The gut microbiome has been implicated in cancer progression and immunotherapeutic responsiveness of different cancers [4,5], and emerging evidence suggests that the female reproductive tract microbiome may play a similar role, particularly in gynecologic malignancies. Cancers of the female reproductive tract account for more than 100,000 new cancer diagnoses and up to 30,000 deaths per year in the United States [6,7,8,9]. Gynecologic malignancies in which microbiomes are currently under study encompass cancers of the lower female reproductive tract, particularly the uterine cervix, as well as the ovary and endometrium in the upper female reproductive tract.

Recent studies applying next-generation sequencing of the bacterial 16S ribosomal RNA (16S rRNA) gene have provided convincing evidence for the existence of an upper female reproductive tract microbiome. Over the past few years, studies of endometrial and peritoneal samples obtained from healthy women and those with benign and noninfectious gynecologic conditions suggest a microbiota continuum with decreasing biomass and increasing diversity from the lower to upper female reproductive tract [10,11]. Data on upper female reproductive tract microbiome composition and its association with gynecologic cancers can be considered preliminary at present. Further studies including multi-omics and systems biology approaches are required to determine functional relevance rather than pure presence of different microbiota and to establish causative relationships with gynecologic diseases including cancer.

## 2. The Lower Female Reproductive Tract (FRT) and Vaginal Microbiome

The mucosal layer of the lower female reproductive tract (FRT) contains commensal bacteria that play important roles for mucosal health. The vaginal microbiome is commonly composed of different species of *Lactobacillus*, which can include *L. crispatus*, *L. gasseri*, *L. iners*, *L. jensenii*, and others [12]. A *Lactobacillus*-dominant vaginal microbiome is considered to be favorable due to the protective characteristics that are provided by these bacterial communities. This includes the production of lactic acid to lower the vaginal pH and the production of bacteriocins and hydrogen peroxide providing an acidic and antimicrobial environment unfavorable for invading pathogens [13,14]. Nevertheless, different *Lactobacillus* species vary considerably in their ability to produce lactic acid and antimicrobial factors, and several are associated with increased mucosal inflammation [15,16]. Molecular profiling has increased the understanding of vaginal microbial diversity, which has been classified into so-called community-state types as defined by the predominant taxa [12]. Often, *Lactobacillus* is not the dominant microorganism, and many women have higher diversity non-*Lactobacillus* microbiota characterized by an overgrowth of obligate and facultative anaerobes such as *Gardnerella*, *Prevotella*, *Atopobium*, and *Mobiluncus*, which is known as microbial dysbiosis or bacterial vaginosis. Vaginal microbial dysbiosis is associated with changes to the mucosal microenvironment, including increased pro-inflammatory cytokines [17], increased numbers of immune cells such as CD4+ T-cells [13,18] that are activated, altered immune pathways [18,19,20,21], epithelial barrier disruption [22], and production of immunomodulatory metabolites [23,24]. Vaginal microbial dysbiosis is associated with numerous adverse reproductive health outcomes, including increased acquisition of sexually transmitted infections, increased risk of pre-term birth, and increased risk of HPV infection and subsequent cervical cancer, among others [25,26,27,28,29,30]. Bacterial vaginosis is the most common vaginal condition for women of reproductive age [31], affecting 23–29% of women globally [32] and causing detrimental effects on a woman’s quality of life physically, emotionally, sexually, and socially [33]. The association between highly polymicrobial non-*Lactobacillus* dominant vaginal microbiome profiles with increased risk of HPV infection, persistence, delayed clearance, and increased risk of developing HPV-associated cervical neoplasia was demonstrated in multiple studies [28,29,30] and was recently reviewed elsewhere [34].

## 3. The Upper Female Reproductive Tract (FRT) Microbiome and Gynecologic Cancers

Studying the upper FRT and specifically the intrauterine and endometrial microbiome is hampered by challenges of sterile access without cervicovaginal contamination and the significantly lower biomass of the upper compared to the lower FRT. These challenges in studying upper FRT microbiota and variability in reported microbiome composition were also discussed in a prior review article and commentary [35,36,37]. A limited number of recent studies, summarized in Table 1, have analyzed the presence and composition of a possible uterine and upper FRT microbiome using either transcervical access to the uterine cavity, or sterile access following sterile transection of surgical hysterectomy specimens or during elective caesarean section. Transcervical endometrial specimens included sampling of catheter-tips following embryo transfer for in vitro fertilization (IVF) [38,39], aspiration of endometrial fluid [40], endometrial swabs [41,42,43], or transcervical tissue sampling by endometrial biopsy or curettage [41,44,45]. Approaches attempting sterile endometrial sampling to avoid passage of the endocervix used sterilely opened hysterectomy specimens from women with benign uterine disease or endometrial cancer to collect endometrial swabs [10,11,46] sometimes with concurrent endometrial tissue samples [47,48,49,50] or endometrial tissue samples obtained at the time of elective caesarean section [51] (see Table 1).

Published reports to date suggest that the upper FRT shares some bacterial species with the lower FRT, which may act as a reservoir for both healthy commensal or pathogenic populations [11,50]. Additional possible routes of transmission and microbial seeding of the uterine cavity include hematogenous spread of gut and oral microbiota; oral and perianal mucosa of an individual and their sexual partners; and microbiota in semen [35,52,53,54,55,56].

Although the vaginal microbiome is dominated by *Lactobacillus* species in the majority of women, it is unclear if this characteristic is shared in healthy or disease states of the upper FRT including the endocervical canal and uterine cavity. While most studies of transcervically obtained intrauterine samples found a dominance of *Lactobacillus* [38,39,40,41,42,44,45], the presence and relative abundance of *Lactobacillus* in uterine samples was more variable when obtained from sterilely opened hysterectomy specimens [10,11,46,47,48,49,50]. Several studies that compared lower and upper FRT microbiomes from the same individual and endometrial samples which were not collected transcervically have suggested decreasing abundance of *Lactobacillus* from the lower to upper FRT microbiomes. These studies found greater diversity in the uterine cavity and greater abundance of species other than *Lactobacillus* both in states of health and malignancy [11,44,46,48]. Despite some compositional overlap between lower and upper FRT, functional analyses of the upper FRT microbiome have not yet been reported, and it is unknown whether the same bacterial species may have distinct interactions with the mucosa of the endometrium compared to the vagina or cervix.

The immunological environment of the endometrium and its ability to respond to various pathogens and to modulate immune responses has been demonstrated in the context of embryo implantation, infertility treatments, and reproductive health outcomes [57,58]. The importance of host immune responses within the endometrium has long been recognized for immune tolerance to fetal antigens and to allow trophoblast invasion and vascular remodeling during implantation. In addition, there is an emerging notion that host immune responses in the endometrium may be further modulated by the existence of an upper FRT and uterine microbiome [57,58]. Future studies investigating the functional relationship between the microbiome, mucosal inflammation, and host immune responses within the upper FRT and uterine cavity in the context of pregnancy and gynecologic cancers may inform each other and ultimately lead to personalized interventions.

### 3.1. Endometrial Cancer and the FRT Microbiome

Endometrial adenocarcinoma is the most common cancer of the female reproductive tract in the Western world, with an estimated 60,000 new cases and 12,000 deaths in the United States in 2021 [18]. As the US population undergoes demographic changes leading to increased life expectancy and higher prevalence of key risk factors such as obesity, the incidence of endometrial cancer is rising and a disproportionate increase in aggressive non-endometrioid subtypes is projected to worsen outcomes. Endometrial cancer is increasingly appreciated to represent a heterogeneous group of cancer subtypes that share the endometrium as an organ of origin but are driven by diverse molecular mechanisms. The Cancer Genome Atlas (TCGA) established four molecular subtypes of endometrial cancer with prognostic relevance [61,62]. The presence and extent of anti-tumor immune responses have emerged as strong prognostic and predictive markers in solid malignancies, including endometrial cancer [63,64]. However, little is known about how this relates to tumor immunology, the upper FRT mucosal microenvironment, and resident microbiome. The majority of studies on the upper FRT microbiome have been conducted in healthy women or those with benign uterine disease such as endometriosis or uterine fibroids, and only three published studies to date reported on the FRT microbiome of women with endometrial cancer compared to healthy controls [47,49,50].

In 2016, Walther-António et al. reported a first cross-sectional study from a US tertiary care center including 31 women undergoing hysterectomy for endometrial cancer (n = 17), endometrial hyperplasia (n = 4), or benign uterine disease (n = 10). The authors found a higher degree of FRT microbial diversity in women with endometrial cancer compared to that of healthy controls, and enrichment of particular taxa such as Firmicutes, Spirochaetes, Actinobacteria, Bacteroidetes, and Proteobacteria [50]. A subsequent larger study by the same group comprised 148 women, including 66 with endometrial cancer, 7 with atypical endometrial hyperplasia, and 75 with benign uterine disease, and represents the largest study of the FRT microbiome in endometrial cancer to date. The authors confirmed their earlier findings of increased diversity of the upper FRT microbiome in patients with endometrial cancer compared to that of healthy controls [49]. Interestingly, the authors found that changes in lower FRT rather than upper FRT microbiome profiles demonstrated the strongest association with endometrial cancer. In addition, FRT microbiome changes were related to other risk factors of endometrial cancer, such as age, menopausal status, and obesity [49].

A recent study in a Chinese cohort of 50 women undergoing surgery for benign uterine disease (n = 25) or endometrial cancer (n = 25) reported some conflicting findings [47]. Lu et al. detected similar phyla in greater abundance in the upper FRT of endometrial cancer patients compared to those with benign uterine disease, including Proteobacteria, Actinobacteria, Firmicutes, and Bacteroidetes, with *Micrococcus* being most significantly increased in endometrial cancer at the genus level. However, in contrast to the two earlier studies by Walther-António and colleagues, they reported a lower diversity of FRT microbiomes in association with endometrial cancer. This study also reported increased gene expression of three inflammatory cytokines, IL-6, IL-8, and IL-17, in endometrial tissues from cancer patients compared to patients with benign disease, and a positive correlation between cytokine expression and the relative abundance of *Micrococcus* [47]. Observations in a single endometrial cancer cell line in vitro suggest that co-culture with *Atopobium* spp. and *Porphyromonas spp*. can induce expression of proinflammatory cytokines in HEC-1A endometrial cancer cells [65], warranting further investigation. Findings that microbial populations in the vagina and lower FRT are related to factors long known to be associated with endometrial cancer risk such as menopausal status [66,67,68], obesity [69], pelvic inflammatory disease [70], and race [71,72,73,74,75] further support a correlative, if not yet causative, relationship between changes in the FRT microbiome and the development of endometrial cancer.

In summary, the existing literature suggests an association between the FRT microbiome and endometrial cancer, but it does not yet allow conclusions about whether this may represent cause or consequence. Future studies providing causative and mechanistic insight into the functional relationship between FRT microbiome and endometrial cancer development and progression will be critical in understanding how we may leverage this knowledge for personalized medicine including FRT microbiome-targeted interventions.

### 3.2. Ovarian Cancer and the FRT Microbiome

Among all gynecologic cancers, the least data exists on the relationship between ovarian cancer and the FRT microbiome. Ovarian cancer is the least common but most lethal of all gynecologic cancers with 21,000 newly diagnosed cases and 14,000 deaths each year in the United States and >150,000 deaths worldwide each year [76]. The lack of effective screening tests and non-specific symptoms contribute to diagnosis at later stages and poor outcome [77]. The peritoneal cavity, where ovarian cancer arises and spreads, has long been considered a sterile environment under physiologic conditions, but this view has recently been challenged. There is emerging evidence of microbial colonization of the peritoneum and correlation between the peritoneal and upper and lower FRT microbiome within an individual [11]. This suggests that FRT health and dysbiosis may influence inflammation and immune response within the peritoneal cavity and consequently may affect development and progression of cancers arising and spreading in the peritoneal cavity, one of the most common being ovarian cancer.

A recent ovarian cancer case-control study of 580 European women assessed the cervicovaginal microbiome in cytological ThinPrep samples by 16S rRNA sequencing [78]. This study cohort included 176 ovarian cancer patients, 109 women without cancer but with known BRCA1 mutations, and 295 healthy controls from the United Kingdom, Italy, Germany, Norway, and the Czech Republic. The authors found that the presence of ovarian cancer and known risk factors including BRCA1 mutation and older age were significantly associated with non-*Lactobacillus* dominant cervicovaginal microbiomes [78]. Limitations of this study include the lack of microbiome analysis of the upper FRT, where ovarian cancer originates, and the use of ThinPrep samples commonly obtained in a methanol-based fixative, which may have altered the results of microbiome analysis.

Zhou et al. [60] studied the intratumoral ovarian cancer microbiome by 16S rRNA sequencing in 25 fresh tumor samples of high grade serous ovarian cancer compared to 25 normal distal fallopian tubes from healthy women. The authors found a similar microbial abundance but slightly decreased diversity in ovarian cancer versus normal tissue. Proteobacteria and Firmicutes were the most frequent taxa identified in both tumor and normal samples, with differences in the relative proportions of certain phyla. At the genus level, this study observed an enrichment of *Acinetobacter* in ovarian cancer and enrichment of *Lactococcus* in normal fallopian tube tissues [60]. This study further supports the presence of an upper FRT microbiome in sterilely collected samples from ovaries and fallopian tubes. However, the descriptive nature of the study does not allow conclusions about whether changes in bacterial abundance and composition are a cause or consequence of ovarian cancer development. One study of routine formalin-fixed and paraffin-embedded archival pathological tissues found distinct viral and bacterial profiles in ovarian cancer compared to those of matched and non-matched normal controls; however, the use of non-sterilely collected paraffin-embedded samples holds the potential for sample contamination, and the use of a microarray platform primarily focused on viral detection and including only probes for 320 bacteria provides only limited insight into tumor microbial profiles [79]. A small study found decreased microbial diversity and a unique microbial profile in the peritoneal fluid of 10 ovarian cancer patients compared to 20 patients with benign ovarian lesions [80]. A set of 18 microbial organizational taxonomic units was enriched in women with ovarian cancer, including bacteria of gut microbial origin and with predicted functionality centered around inflammatory mediators. Interestingly, the addition of peritoneal microbial features increased the accuracy of common serum tumor markers such as CA-125 for the diagnosis of ovarian cancer in this limited cohort of 30 patients [80].

Despite the current lack of functional microbiome analysis and established causative relationships, the observed associations between FRT microbial dysbiosis and ovarian cancer risk warrant further research exploring FRT and peritoneal microbiome as potential adjuncts to ovarian cancer prevention, diagnosis, and treatment.

## 4. Interventions Targeting the FRT Microbiome and Implications for Personalized Medicine

The FRT microbiome has been demonstrated to be modifiable through lifestyle factors, including diet, smoking, exercise, hygiene, and sexual practices, and hormone status [81,82,83]. The loss of estrogen in the vaginal epithelium either from natural menopause or the results of hormonal or cytotoxic cancer therapies induces a shift towards non-*Lactobacillus* dominant lower FRT microbiomes with increased abundance of anaerobes such as *Gardnerella* and *Atopobium*. In addition, there is evidence that other body sites including oral and perianal mucosa [52,53,54] and sexual partners [52,84] may act as a reservoir for the FRT microbiome, which needs to be taken into consideration when designing interventions. To date, interventions manipulating the FRT microbiome have not yet been explored in the setting of gynecologic cancers, and therapies targeting the lower FRT microbiome have been focused on treatment of vaginal microbial dysbiosis/bacterial vaginosis. Nevertheless, experiences from treating bacterial vaginosis may provide a proof of principle for designing future interventions modulating the FRT microbiome in the context of prevention or treatment of gynecologic cancers.

### 4.1. Treatment of Vaginal Microbial Dysbiosis or Bacterial Vaginosis

Treatment of bacterial vaginosis (BV) is mostly limited to antibiotic options, including metronidazole or clindamycin, which lead to only a transient resolution, with approximately 50% of women having a recurrence within one year post-treatment [85]. The promotion of growth of *Lactobacillus* species in the vaginal tract may be multifaceted and include the removal of BV associated bacteria, but also the degradation of the protective biofilm formation by BV-associated bacteria such as *Gardnerella vaginalis*, or the presence and promotion of sufficient *Lactobacillus* bacteria to generate lactate or hydrogen peroxide that will decrease the mucosal pH. Other treatment options have been explored in clinical practice in addition to antibiotic treatment, such as using boric acid [86,87] or probiotics/prebiotics that promote a more favorable vaginal microbiome composition [88]. Boric acid has been used in a few clinical trials to treat recurrent BV and was found to be well tolerated as an intravaginal application [86,89]. The mechanism by which it alleviates symptomatic BV is thought to be through the inhibition of biofilm formation, which may decrease host inflammation in the mucosal site, promoting epithelial repair. The presence of biofilms may also decrease the ability of host antibodies to reach BV-associated bacteria, and therefore decreasing this physical barrier may promote antibody activity.

Numerous different probiotics are available for use to the public that are advertised as a treatment for BV, and clinical studies have shown some success using oral or vaginal probiotics to treat recurrent BV [88,90,91]. The addition of probiotics may promote *Lactobacillus* stability, increasing the presence of immunomodulatory products such as hydrogen peroxide and lactic acid, decreasing inflammation. Recently, there have been studies evaluating live biotherapeutic products [92,93] or vaginal microbiome transplants [94] as therapeutic options for recurrent BV. The first vaginal microbiome-based live biotherapeutic product, named Lactin-V, uses a strain of *Lactobacillus crispatus* isolated from the vagina of a healthy woman [93]. In a randomized, double-blind, placebo-controlled phase 2b trial, recurrent BV was found in 30% of women in the Lactin-V treated group and 45% in the placebo group after 12 weeks, and there were no adverse effects recorded to be greater in the treated group than the placebo group. The first vaginal microbiome transplant was performed on 5 patients, with 4 observing long term remission (21 months) after treatment [94]. No adverse effects were observed in this study. However, three patients required repeated microbiota transplant procedures to work effectively, and one had to use a different donor to achieve a stable *Lactobacillus* vaginal microbiome after a second treatment. This indicates vaginal microbiome transplants are a complex treatment option that would be personalized on a case-by-case basis and not necessarily appropriate for a global treatment option.

Lessons from treating vaginal microbial dysbiosis suggest that maintenance of a healthy FRT microbiome is complex, and future interventions may require combinatorial approaches targeting the microbiota along with the mucosal microenvironment including immunomodulatory microbial metabolites and host immune response to achieve a lasting therapeutic modulation of the FRT microbiome.

### 4.2. FRT Microbiome and Cancer Therapy

There are no published studies yet demonstrating an impact of the FRT microbiome on response to treatment of gynecologic cancers. Changes in the vaginal and cervical microbiome were observed following pelvic radiation therapy, a commonly used primary or adjuvant treatment for cervical and endometrial cancer [95,96]. However, possible associations between the FRT microbiome and treatment response were not assessed in these small pilot studies.

Associations have been recently reported between antibiotic treatment and the outcome of women with gynecologic cancers undergoing chemotherapy or immunotherapy. In a retrospective study of 424 women with advanced stage epithelial ovarian cancer who underwent surgery and platinum-based chemotherapy, receiving antibiotic treatment against gram-positive organisms for at least 48 h during chemotherapy was associated with decreased progression-free and overall survival [97]. Similarly, in a retrospective study of 101 recurrent endometrial, ovarian, and cervical cancer patients, pre-treatment with antibiotics within the 30 days prior to treatment, but not during treatment, with immune checkpoint inhibitors was associated with decreased response rate and shorter progression-free and overall survival [98]. Further studies are needed to validate the adverse effect of antibiotic treatment on response to cancer chemotherapy or immunotherapy, its dependence on timing relative to cancer therapy, and whether its effect is mediated through changes in the gut or FRT microbiome or both.

Increasing evidence has implicated the gut microbiome in modulating the response to cancer immunotherapies [4,5], and early phase clinical trials have demonstrated successful gut microbiome-targeted interventions to improve treatment response. Therapeutic modulation of the gut microbiome such as bacterial supplementation with *Akkermansia* [99] or fecal microbiota transplants derived from donors with prior favorable response to cancer immunotherapy [100] have been shown to overcome resistance to checkpoint inhibitors such as anti-PD-1 therapy in previous non-responders. These studies also found unique proteomic and metabolomic signatures in the tumor microenvironment, suggesting the effect is therapeutically transferrable [100].

Together, these prior reports demonstrating the importance of the gut microbiome for modulating responses to cancer treatment including chemotherapy and immunotherapy provide a rationale for exploring similar effects of the FRT microbiome in future studies.

## 5. Perspective and Future Directions

We deliberately focused our review on microbiome data from human cohorts, and a discussion of FRT microbiome of in vivo or in vitro models of gynecologic cancers was beyond the scope of this article. In conclusion, we have discussed the evidence supporting the existence of an upper FRT and uterine microbiome and emerging evidence suggesting its interplay with local inflammation and host immune responses. Despite the current lack of mechanistic insight, observed associations between changes in the FRT microbiome and the development, progression, and outcome of gynecologic cancers warrant further studies. Evidence demonstrating the importance of the gut microbiome for response to cancer treatments and early success of gut microbiome-targeted interventions lay the groundwork to explore FRT microbiome-targeted interventions. The FRT microbiome has been demonstrated to be modifiable through nutritional interventions, probiotic and live biotherapeutic products, and vaginal microbiota transplants. If causative relationships to carcinogenesis can be established, microbiome-targeted interventions might aid in primary and secondary preventions of gynecologic cancers or be able to modulate and enhance responses to conventional cancer therapies. Future studies should include multi-omics approaches including metagenomics, metaproteomics, and metabolomics for functional analysis of the FRT microbiome to gain mechanistic insight and identify targets for personalized medicine.

## Figures and Tables

**Table 1:** Summary of studies analyzing the upper FRT microbiome, including uterine cavity and endometrium, ovaries, fallopian tubes, and peritoneal cavity.

Year and First Author	Study Subjects	Study Region	Microbial Analysis	Sampling Method	Dominant Uterine Bacterial Organisms	Main Findings
2015 Mitchell [10]	58 women, mean age 43 years, undergoing surgery for benign uterine disease	United States	qPCR for 12 species-specific 16S rRNA genes	**Sterile** collection of uterine and endocervical swabs at time of surgery, surgeries completed without intracavitary uterine manipulator	Species: -*Lactobacillus iners* (45%) -*Lactobacillus crispatus* (33%) -*Prevotella* spp. (33%)	The upper FRT is not sterile. Upper FRT microbiome is lower in biomass than is the lower FRT.
2016 Fang [41]	30 reproductive age women with regular menses with or without endometrial polyps	China	16S rRNA Seq (V4)	Transcervical endometrial swabs and **tissue** sampling using cervicovaginal prep and vaginal sleeve	Genera: -*Lactobacillus* (26%) -*Enterobacter* (16%) -*Pseudomonas* (13%)	The uterine cavity is not sterile. Differences in population may associate with the presence of endometrial polyps.
2016 Franasiak [38]	33 reproductive age women	United States	16S rRNA Seq (V2-3)	Transcervical IVF catheter-tip sampling	Genera: -*Flavobacterium* -*Lactobacillus*	*Flavobacterium* and *Lactobacillus* dominate the uterine cavity microbiome at time of embryo transfer.
2016 Khan [42]	32 reproductive age women with and without endometriosis	Japan	Targeted 16S rRNA Seq for 58 species (V3)	Transcervical endometrial swabs	Family: -Lactobacillacae -Streptococcaceae -Staphylococaceae -Enterobacteria-ceae -Moraxellaceae	The uterine cavity is not sterile. Populations may vary between patients with and without endometriosis.
2016 Moreno [40]	70 reproductive age women (3 independent pilot cohorts: n = 13 and n = 22 fertile and n = 35 infertile women)	Spain	16S rRNA Seq (V3-V5)	Transcervical endometrial fluid sampling (aspiration of cervical mucus prior to endometrial sampling to reduce contamination)	Genera: -*Lactobacillus* (72%) -*Gardnerella* (13%) -*Bifidobacterium* (4%) -*Streptococcus* (3%) -*Prevotella* (0.9%)	The uterine cavity is not sterile. Endometrial and vaginal microbiota differ compositionally in some women. Non-*lactobacillus* dominant endometrial microbiota may be associated with negative reproductive outcomes in IVF patients.
2016 Verstraelen [43]	19 women of reproductive age	Belgium	16S rRNA Seq (V1-2)	Transcervical sampling with Tao brush with cervicovaginal sheath	Phyla: -Bacteroidetes -Proteobacteria -Firmicutes	The uterine cavity is not sterile.
2016 Walther-António [50]	31 women undergoing hysterectomy for benign or malignant uterine disease	United States	16S rRNA Seq (V3-5)	**Sterile** collection of uterine swabs and **tissue** at time of surgery	Genera: -*Shigella* -*Barnesiella* -*Staphylococcus* *-Blautia* *-Parabacteroides* *-Bacteroides* *-Faecalibacterium*	The uterine cavity is not sterile. Specific bacteria present in the upper FRT may be associated with gynecologic malignancy.
2017 Chen [11]	95 reproductive age women undergoing hysterectomy for benign uterine disease not known to involve infection	China	16S rRNA Seq (V4-5)	**Sterile** collection of swabs from uterine cavity at time of surgery (additional vaginal and cervical swabs obtained prior to surgery)	Genera: -*Lactobacillus* (31%) -Others (11%) -*Acinetobacter* (9%) -*Pseudomonas* (9%)	The uterine cavity and upper FRT are not sterile. There is a continuum of microbiota from the lower to upper FRT with decreasing biomass, decreasing *Lactobacillus* abundance, and increasing diversity towards upper FRT.
2017 Miles [48]	10 women undergoing hysterectomy and bilateral salpingo-ophorectomy for benign (n = 8) or malignant (n = 2) conditions	United States	16S rRNA Seq (V1-3)	**Sterile** collection of **tissue** and swabs from uterine cavity at time of surgery	Genera: -*Lactobacillus* -Other *-Corynebacterium* *-Staphylococcus* *-Blautia*	The uterine cavity and upper FRT are not sterile.
2017 Tao [39]	70 women undergoing IVF embryo transfers	United States	16S rRNA Seq (V4)	Transcervical IVF catheter-tip sampling	Genera: -*Lactobacillus* -*Corynebacterium* -*Bifidobacterium* -*Staphylococcus* -*Streptococcus*	IVF catheter-tip sampling provides data on the low biomass uterine microbiome.
2018 Liu [59]	25 women with recurrent pregnancy loss treated at IVF clinic	China	16S rRNA Seq (V4)	Transcervical sheathed catheter endometrial fluid and **tissue** sampling	Genera: *-Lactobacillus* *-Gardnerella* *-Atopobium* *-Bifidobacterium*	The uterine cavity is not sterile. Endometrial fluid sampling may not fully reflect microbiome composition in endometrial tissue.
2018 Wee [45]	31 women undergoing hysteroscopy for benign indications, with and without history of infertility	Australia	16S rRNA Seq (V1-V3)	Transcervical endometrial **tissue** biopsy (curette)	Genera: -*Lactobacillus* -*Bifidobacterium* *-Corynebacterium* *-Gardnerella* -*Propionibacterium* *-Propionimicrobium*	The uterine cavity is not sterile. Bacterial abundances differ between the upper and lower FRT, and composition may differ by fertility status.
2019 Walsh [49]	148 women undergoing hysterectomy for benign disease, endometrial hyperplasia, or endometrial cancer	United States	16S rRNA Seq (V3-5)	**Sterile** collection of uterine swabs and **tissue** at time of surgery	Phyla: -Firmicutes -Bacteroidetes -Actinobacteria	The uterine cavity is not sterile. The FRT microbiome is significantly different between patients with and without endometrial cancer. The lower and upper FRT microbiome are correlated.
2019 Winters [46]	25 pre-menopausal women undergoing hysterectomy for benign uterine disease	Italy	16S rRNA Seq (V4); and universal and *Lactobacillus*-targeted 16S rRNA qPCR	**Sterile** collection of uterine swabs at time of surgery. Additional collection of vaginal, cervical, rectal, and oral swabs	Genera: -*Acinetobacter* *-Pseudomonas* *-Cloacibacterium* *-Comamonadaceae*	The uterine cavity is not sterile. The uterine microbiome most closely resembles the cervical microbiome but differs from the vaginal microbiome and is not dominated by *Lactobacillus*.
2019 Zhou [60]	25 women with ovarian cancer and 25 women undergoing salpingo-oophorectomy for benign uterine disease	China	16S rRNA Seq (V3-4)	**Sterile** collection of ovarian cancer and normal distal fallopian tube tissue at the time of surgery	Not assessed	The upper FRT is not sterile. Proteobacteria and Firmicutes are the most frequent taxa in ovarian cancer and normal fallopian tubes, with differences at the phylum/genus level.
2019 Leoni [51]	19 women undergoing elective caesarean section for normal pregnancies at full term.	Italy	16S rRNA Seq (V5-6)	**Sterile** collection of endometrial tissue biopsies during elective caesarean section	Genera: -*Propionibacterium* *-Escherichia* *-Staphylococcus* *-Acinetobacter* *-Streptococcus*	The uterine cavity of term pregnancies is not sterile. *Lactobacillus* was not abundant in the majority of subjects.
2020 Riganelli [44]	34 reproductive age women undergoing infertility treatment	Italy	16S rRNA Seq (V3-4)	Transcervical endometrial **tissue** biopsy (pipelle) using a vaginal and cervical sheath	Phyla: -Firmicutes -Proteobacteria -Bacteroidetes -Actinobacteria	The uterine cavity is not sterile. The uterine microbiome is compositionally different from the vaginal microbiome.
2021 Lu [47]	50 women undergoing hysterectomy for benign disease or endometrial cancer	China	16S rRNA Seq (V3-4)	**Sterile** collection of uterine swabs and **tissue** at time of surgery, noted sterile instruments used	Genera: -*Rhodococcus* *-Phyllobacterium* *-Sphingomonas* *-Bacteroides* *-Bifidobacterium*	The uterine cavity is not sterile. The uterine microbiome differed in diversity and composition between endometrial cancer and benign disease.

16S rRNA Seq, sequencing of bacterial 16S ribosomal rRNA genes, with primer sets used for variable gene regions indicated in parentheses; qPCR, quantitative PCR; IVF, in vitro fertilization.

## Data Availability

Not applicable.
